# Strategic Objectives of Nanotechnology-Driven Repurposing in Radiopharmacy—Implications for Radiopharmaceutical Repurposing (Beyond Oncology)

**DOI:** 10.3390/pharmaceutics17091159

**Published:** 2025-09-03

**Authors:** María Jimena Salgueiro, Marcela Zubillaga

**Affiliations:** 1 Departamento de Física, Facultad de Farmacia y Bioquímica, Universidad de Buenos Aires, Buenos Aires 1113, Argentina; mzubi@ffyb.uba.ar; 2 Instituto de Tecnología Farmacéutica y Biofarmacia (InTecFyB), Universidad de Buenos Aires, Buenos Aires 1113, Argentina; 3 Consejo Nacional de Investigaciones Científicas y Técnicas (CONICET), Buenos Aires 1113, Argentina

**Keywords:** nanotheranostics, drug repurposing, multifunctional nanoparticles, hybrid imaging agents, combination therapies, radiopharmaceuticals, regulatory frameworks, personalized medicine, clinical translation, AI-assisted treatment planning

## Abstract

The integration of nanotechnology into drug repurposing strategies is redefining the development landscape for diagnostic, therapeutic, and theranostic agents. In radiopharmacy, nanoplatforms are increasingly being explored to enhance or extend the use of existing radiopharmaceuticals, complementing earlier applications in other biomedical fields. Many of these nanoplatforms evolve into multifunctional systems by incorporating additional imaging modalities (e.g., MRI, fluorescence) or non-radioactive therapies (e.g., photodynamic therapy, chemotherapy). These hybrid constructs often emerge from the reformulation, repositioning, or revival of previously approved or abandoned compounds, generating entities with novel pharmacological, pharmacokinetic, and biodistribution profiles. However, their translational potential faces significant regulatory hurdles. Existing frameworks—typically designed for single-modality drugs or devices—struggle to accommodate the combined complexity of nanoengineering, radioactive components, and integrated functionalities. This review examines how these systems challenge current norms in classification, safety assessment, preclinical modeling, and regulatory coordination. It also addresses emerging concerns around digital adjuncts such as AI-assisted dosimetry and software-based therapy planning. Finally, the article outlines international initiatives aimed at closing regulatory gaps and provides future directions for building harmonized, risk-adapted frameworks that support innovation while ensuring safety and efficacy.

## 1. Introduction

This review does not follow the traditional format of simply listing and summarizing experimental studies or nanotechnology-based formulations already developed for drug repurposing. Instead, it aims to provide a comprehensive and integrative perspective that synthesizes, through an exhaustive review of the literature, the multiple and often interrelated ways in which nanotechnology can support and enhance the repurposing of radiopharmaceuticals.

In this review, drug repurposing in radiopharmacy encompasses both of the following:

(i) The reformulation, repositioning, or revival of radiolabeled compounds already approved for clinical use, in order to expand their indications, improve their safety profile, or enhance their diagnostic/therapeutic performance; 

(ii) The identification of new clinical applications for radiopharmaceuticals that have reached advanced preclinical stages but were not previously translated into routine practice—often due to limitations in biodistribution, stability, or regulatory feasibility that can be overcome through nanotechnology-enabled strategies.

This dual perspective allows us to address opportunities for clinical expansion of established agents as well as the rescue of promising but underutilized preclinical radiopharmaceuticals.

The manuscript focuses on conceptual and strategic contributions—such as improved pharmacokinetics, targeted delivery, and modulation of biological interactions—that nanotechnology offers to reposition known radiotracers or therapeutic radionuclides for new diagnostic or therapeutic applications. By structuring the discussion around key technological and translational dimensions, this work seeks to identify patterns, gaps, and opportunities rather than merely report existing outcomes.

A radiopharmaceutical combines a radionuclide that emits imageable and/or therapeutic radiation with a carrier molecule that delivers the radionuclide to the target, though in some cases, the primary radionuclide (e.g., Na^131^I) itself is responsible for both targeting and irradiation [1]. For imaging, the radionuclide should emit either positrons for positron emission tomography (PET) or photons for single-photon emission computed tomography (SPECT). For radiopharmaceutical therapy, radionuclides that emit ß−particles (electrons) have proven highly effective in clinical practice [2,3,4]. In addition, isotopes that produce α particles [5,6,7]—emissions capable of depositing extremely high amounts of energy across their short path-lengths—have been used clinically (e.g., ^223^RaCl_2_) and have shown promise in advanced clinical trials (e.g., ^225^Ac-DOTA-TATE and ^225^Ac-PSMA-617). Finally, radiopharmaceuticals containing Auger-electron-emitting nuclides remain predominantly in the preclinical arena, though a handful of centers have translated them to the clinic [8].

**Figure 1:**
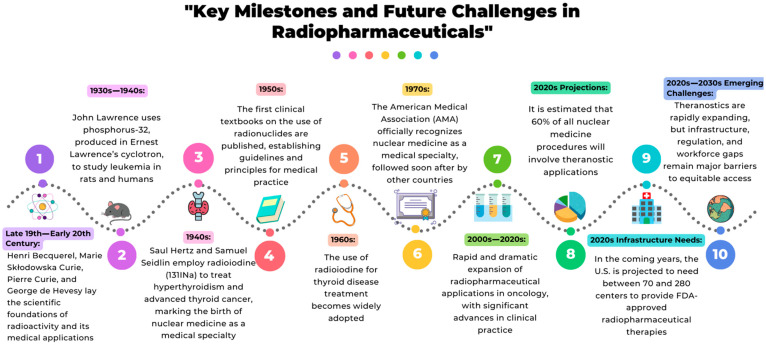
The timeline highlights the major historical breakthroughs that shaped radiopharmaceutical evolution, from the early scientific discoveries to its rapid expansion. It also outlines current projections and emerging challenges, emphasizing the need for infrastructure, regulatory development, and global workforce training to ensure equitable access to advanced medical technologies that employ radiopharmaceuticals.

In 2002, Funkhouser et al. introduced and defined the term ‘theranostics’ as a single compound containing both diagnostic and therapeutic properties [9], although a growing number of these radiopharmaceuticals are in fact labeled with ‘theranostic pairs’ of radionuclides that facilitate the use of closely related probes for both imaging and therapy. Theranostics was introduced as a new field in medicine called ‘personalized medicine’ or ‘personalized therapy’, and Hick summarized this field in one sentence as “if you can see it, you can treat it” [10] (Figure 1). The use of radiopharmaceuticals in various diseases like cancer, cardiovascular, endocrine, and neurological disorders is increasing day by day and, therefore, gaining more attention from scientists in the modern healthcare system. Over the past two decades, the clinical use of radiopharmaceuticals—especially theranostics—has surged dramatically. Market forecasts project that within ten years, approximately 60% of nuclear medicine procedures will involve theranostics, and that the U.S. will require up to 280 dedicated centers to meet clinical demand. Simultaneously, global revenues are expected to grow from USD 11.9 billion (2024) to over USD 35 billion by 2034, underscoring escalating pressures on manufacturing, distribution, and workforce infrastructure [11,12].

Currently, radiopharmacy faces several challenges in the development of radiopharmaceuticals, such as manufacturing and production difficulties, regulatory and reimbursement barriers, access and availability of radionuclides, integration into clinical practice, interdisciplinary collaboration and workforce training, as well as scientific and technological innovation [13].

Radiopharmaceuticals in nuclear medicine continue to face persistent challenges, primarily due to their limited and often non-uniform uptake within target tissues such as solid tumors. These limitations have historically led to the rejection or underutilization of promising radiopharmaceutical candidates, despite their potential diagnostic or therapeutic value. Nanotechnology offers an opportunity not only to enable the development of novel delivery systems but also to reposition previously discarded radiopharmaceuticals and to enhance the performance of those already established in clinical practice. While oncology remains a primary focus due to the high incidence of solid tumors (e.g., sarcomas, carcinomas, and lymphomas account for over 85% of all human cancers), the benefits of radiopharmaceuticals—sensitive diagnostic signals and the ability to induce targeted cell death—extend far beyond cancer. Fields such as cardiology, neurology, and infectious disease could greatly benefit from improved delivery platforms that optimize biodistribution and minimize systemic toxicity. Therefore, the advancement of nanotechnology-driven radiopharmaceutical delivery strategies capable of overcoming biological barriers, enhancing targeting, and broadening clinical applications is of critical importance [14,15,16,17,18,19,20,21,22,23,24,25,26].

As with many conventional pharmaceuticals, radiopharmaceuticals face challenges related to biodistribution, safety, and therapeutic effectiveness. Nanotechnology has already proven capable of optimizing the clinical performance of numerous drugs, either by enhancing their therapeutic effect or by reducing adverse effects through targeted delivery. Radiopharmaceuticals can benefit from these same approaches, achieving improved diagnostic sensitivity and therapeutic outcomes while minimizing off-target exposure. This dual advantage—enhanced efficacy and reduced toxicity—positions nanotechnology as a transformative tool for redefining the role and scope of radiopharmaceuticals across multiple medical specialties [14,15,16,17,18,19,20,21,22,23,24,25,26].

This review aims to provide a comprehensive examination of the evolving role of nanotechnology in drug repurposing within radiopharmacy, with particular emphasis on how nanocarrier systems have contributed, are currently contributing, or may contribute in the future to overcoming key challenges previously outlined. By integrating insights from past studies, current research, and emerging technologies, we highlight how nanotechnology can optimize the clinical performance of both established and previously discarded radiopharmaceuticals.

While previous reviews on radiopharmacy and nanotechnology have primarily focused on cataloguing nanocarrier-based radiopharmaceutical formulations—most of them within the oncology field—this work adopts a different perspective. It systematically frames nanotechnology as a strategic enabler for drug repurposing in radiopharmacy, identifying functional objectives and cross-linking them with macro-level challenges of the discipline. The emphasis is on conceptual and translational strategies, rather than on exhaustive compound listings, and includes non-oncological applications such as cardiology, neurology, infectious and inflammatory diseases. This approach is intended for a multidisciplinary audience—radiopharmacists, nuclear medicine physicians, materials scientists, and regulatory experts—seeking to integrate formulation innovation with repurposing strategies under real-world regulatory and economic constraints (Table 1).

## 2. Methods

Search strategy

A comprehensive search was conducted in PubMed, Scopus, and Web of Science databases from May 2025 to July 2025. The search strategy combined free-text terms and controlled vocabulary (MeSH) related to the main concepts of this review: nanotechnology, radiopharmacy, radiopharmaceutical repurposing, and drug repurposing beyond oncology. The following keywords and Boolean operators were applied:

(“nanotechnology” OR “nanomedicine” OR “nanocarriers” OR “nanoparticles”) AND (“radiopharmacy” OR “radiopharmaceuticals”) AND (“repurposing” OR “drug repositioning” OR “drug reuse”) AND (“beyond oncology” OR “non-oncological applications”).

Additional references were identified through manual screening of the bibliographies of included articles and relevant reviews.

Inclusion and exclusion criteria

Studies were included if they

1. Were published in peer-reviewed journals in English or Spanish.
2. Addressed nanotechnology-based strategies for radiopharmaceutical repurposing, with or without direct clinical application.
3. Focused on non-oncological indications, or provided conceptual/methodological frameworks applicable beyond oncology.

Studies were excluded if they

1. Were conference abstracts without full text.
2. Were duplicates or secondary reports of the same data.
3. Lacked sufficient methodological detail or direct relevance to the topic.

Study selection process

The selection was carried out in two steps:

1. Title and abstract screening to exclude clearly irrelevant records.
2. Full-text review to confirm eligibility according to the predefined criteria.

Quality assessment

Although this is a narrative review, the methodological quality of the included studies was considered based on sample size, experimental design, reproducibility of methods, and clarity in reporting results. When possible, the risk of bias was qualitatively evaluated using adapted domains from the Cochrane Risk of Bias tool (randomization, blinding, completeness of outcome data, and selective reporting).

Handling of conflicting evidence

In cases where studies presented contradictory findings, discrepancies were discussed in light of methodological differences, experimental context, and the robustness of the reported data. The most plausible interpretations were favored based on the overall weight and quality of the evidence.

Flow diagram

The literature selection process followed the general algorithm of the PRISMA guidelines, including database searching, duplicate removal, title/abstract screening, full-text assessment, and final inclusion based on predefined criteria. Numerical data for each step are not presented, as this is a narrative review.

## 3. Current Challenges in Radiopharmacy

Radiopharmacy forms the foundation of nuclear medicine, as neither functional imaging nor targeted radiotherapy would be possible without radiopharmaceuticals [27,28,29,30,31,32,33]. Unlike other imaging modalities, nuclear medicine relies entirely on these compounds for both diagnosis and treatment, underscoring the pivotal role of this discipline. Consequently, nuclear medicine is inherently multidisciplinary, integrating expertise from medicine, chemistry, physics, and biology. The field continues to evolve through the development of innovative diagnostic and therapeutic agents, with theranostic pairs representing the fastest-growing area in line with the rise of personalized medicine and projected to account for the majority of future radiopharmaceutical developments. The timeline presented in this review highlights key milestones in the evolution of radiopharmacy and its central contribution to the emergence of nuclear medicine, providing the context needed to understand the current challenges and future directions of the field [27,34,35,36,37,38,39,40,41].

In recent years, theranostics has emerged as a paradigm shift in radiopharmacy and personalized nuclear medicine, especially in oncology, combining diagnostic imaging and targeted therapy within a single molecular platform. This dual capability enables clinicians to identify, image, and treat tumors with unparalleled precision, effectively applying the concept of ‘seeing what you treat.’ By integrating imaging radiopharmaceuticals to confirm target expression with therapeutic agents delivering localized radiation, theranostics allows real-time monitoring of treatment response while reducing systemic toxicity and the risk of therapeutic failure. Compared to traditional single-purpose diagnostic or therapeutic radiopharmaceuticals, theranostics provides a more efficient route for patient selection, therapy planning, and outcome prediction, ultimately improving treatment efficacy and safety [27,42]. Despite its clinical successes and growing regulatory approvals, significant challenges remain that span the entire value chain, from radionuclide production to clinical translation, and must be addressed to ensure sustainable growth of the field [13,43,44,45,46,47,48,49,50,51,52,53,54,55,56,57,58,59,60,61,62].

To provide a structured overview of the main barriers discussed in this section, Table 2 summarizes the six key challenges in radiopharmaceutical repurposing with nanotechnology, together with representative references and their core implications.

### 3.1. Manufacturing and Production Difficulties

The synthesis and production of radiopharmaceuticals, particularly those involving emerging alpha- and Auger-electron emitters, remain highly complex. Production capacity is often limited to a few specialized facilities, and scaling up these processes while maintaining radiochemical purity and reproducibility is technically demanding. Developing reliable, efficient, and cost-effective manufacturing workflows is critical to meet the growing global demand.

### 3.2. Regulatory and Reimbursement Barriers

Disparities in regulatory requirements and reimbursement policies across countries hinder the broad adoption of novel radiopharmaceuticals. In many regions, global reimbursement frameworks are not adequately designed to cover innovative agents, creating disparities in access and slowing the translation of new technologies into clinical practice.

### 3.3. Access and Availability of Radionuclides

The production of radionuclides depends on a limited global infrastructure. Shortages of key medical isotopes—exacerbated by aging reactor facilities and uneven distribution networks—pose a significant bottleneck for both routine clinical applications and research. Strengthening international collaborations, developing robust online databases for radionuclide availability, and expanding production facilities are essential steps to mitigate these constraints.

### 3.4. Integration into Clinical Practice

Conducting large-scale, randomized clinical trials for radiopharmaceuticals remains challenging due to the complex logistics of isotope supply, ethical considerations related to radiation exposure, and the high costs involved. This slows down the transition from promising preclinical studies to widespread clinical use, particularly for first-line and combination therapies.

### 3.5. Interdisciplinary Collaboration and Workforce Training

Nuclear medicine is intrinsically multidisciplinary, requiring expertise in radiochemistry, pharmacy, imaging technologies, oncology, and medical physics. The rapid pace of innovation demands a highly skilled workforce, yet training programs and collaborative platforms are still insufficient in many regions—particularly in low- and middle-income countries.

### 3.6. Scientific and Technological Innovation

As the field evolves toward more sophisticated theranostic applications, new challenges arise, such as designing advanced chelators for novel radionuclides, improving dosimetry methodologies, and developing delivery systems that ensure both safety and efficacy. These scientific hurdles must be overcome to fully exploit the therapeutic and diagnostic potential of modern radiopharmaceuticals.

Collectively, these barriers slow the realization of radiopharmaceuticals’ full potential in personalized therapy, underscoring the need for innovative strategies, including those offered by nanotechnology, to overcome these hurdles.

Many of the structural and scientific barriers currently limiting the impact of radiopharmaceuticals—such as complex manufacturing, radionuclide shortages, suboptimal biodistribution, translational gaps, regulatory frictions, workforce limitations, and the need for advanced chelation/dosimetry—mirror the challenges faced in conventional drug development. In other pharmaceutical domains, nanotechnology has consistently proven its value by (1) enhancing delivery selectivity, (2) stabilizing labile drugs, (3) enabling multimodal and theranostic applications, and (4) improving the benefit–risk profile. Radiopharmacy can leverage the same technological advances.

Specifically, nanoplatforms can [13]

1. Improve production efficiency and formulation robustness, enhancing stability, scalability, and handling of radioconjugates and nano-radiocarriers.
2. Optimize biodistribution and targeting, reducing off-target effects and increasing the signal-to-background ratio for imaging and therapeutic efficacy.
3. Enhance radiolabeling stability and chelation (especially for emerging radionuclides), reducing radionuclide leaching or recoil effects.
4. Enable multimodal platforms and improved dosimetry by integrating both imaging and therapeutic components within the same nanocarrier, allowing real-time monitoring and precise quantification.
5. Overcome biological barriers (e.g., tumor microenvironment, blood-brain barrier), which have historically limited uniform tumor uptake and led to the abandonment of otherwise promising agents.

From a radiopharmaceutical repurpose, nanotechnology enables two parallel approaches:

(A) Reviving “failed” or underutilized agents whose pharmacokinetics, toxicity, or biodistribution can be optimized through nanoformulation.

(B) Enhancing the clinical performance of well-established radiopharmaceuticals, particularly when pursuing goals such as dose reduction, As Low As Reasonably Achievable (ALARA) compliance, theragnostic pairing, or multimodal imaging.

Within this context, nanocarriers are being explored—or could be explored—to achieve five strategic objectives that directly address the broader challenges outlined above [63]:

(1) Mitigating Medical Isotope Shortages

By improving delivery efficiency and retention in target tissues, nanocarriers allow for lower administered activities without sacrificing diagnostic or therapeutic outcomes, thereby extending limited radionuclide supplies such as ^99^Mo/^99m^Tc [64,65,66,67,68,69,70,71,72,73,74]. 

Key challenges addressed: Manufacturing and production, Radionuclide access, Scientific and technological innovation (through designs that maximize performance per dose). 

Repurposing benefit: Revives compounds previously considered unfeasible due to isotope cost or scarcity.

(2) Reducing Costs Without Upgrading Equipment

While advancements in scanner sensitivity remain important, nano-enabled dose sparing can deliver comparable or superior image quality while reducing isotope waste and operational costs [74,75,76]. 

Key challenges addressed: Manufacturing and production, Reimbursement, Access to radionuclides. 

Repurposing benefit: Enables the reintroduction of radiopharmaceuticals previously limited by economic constraints.

(3) Enhancing Radiation Safety (ALARA Principle)

Selective targeting and improved retention allow for lower patient doses, reduced systemic exposure, and enhanced radiation protection for healthcare workers [74,75,76]. 

Key challenges addressed: Clinical integration (better risk-benefit profile facilitates adoption), Scientific innovation (designing nano-delivery systems with improved PK/PD). 

Repurposing benefit: Rescues radiopharmaceuticals abandoned due to systemic toxicity by improving their therapeutic index.

(4) Enabling Theragnostic Applications

Nanoplatforms enable co-delivery of imaging and therapeutic radionuclides or the integration of theragnostic pairs within a single vector, ensuring synchronized biodistribution and “see what you treat” feedback with real-time dosimetry [77,78,79]. 

Key challenges addressed: Clinical integration (patient selection, monitoring), Scientific innovation (advanced chelation, theragnostic stability), Workforce (requires and fosters multidisciplinary collaboration). 

Repurposing benefit: Converts diagnostic-only radiopharmaceuticals into theragnostic platforms, or vice versa.

(5) Supporting Multimodal Probe Design

Hybrid imaging modalities (PET/CT, PET/MR, SPECT/CT) demand pharmacokinetic compatibility between contrast agents and radiotracers. Nanocarriers enable co-encapsulation or coordination of multiple agents, ensuring harmonized biodistribution and integrable readouts [80,81,82,83,84,85,86,87,88,89,90,91].

Key challenges addressed: Scientific innovation (multimodal design, improved dosimetry), Clinical integration (enhanced data quality).

Repurposing benefit: Positions compounds as building blocks for multimodal imaging platforms, even if they previously lacked clinical impact.

To contextualize how nanotechnology can systematically support radiopharmaceutical repurposing, we mapped five strategic nanotechnology-enabled objectives against six macro-level challenges currently limiting the impact of radiopharmaceutical sciences. This cross-walk highlights where nanocarriers can deliver direct, technically actionable value (e.g., improving dosimetry, stability, and targeting) and where their contribution is indirect but enabling (e.g., easing production pressure or strengthening the case for reimbursement through better cost-effectiveness and safety profiles).

**Table 2:** Overview of current challenges in radiopharmaceutical repurposing using nanotechnology.

Subsection	Challenge	Key Points	Representative References
Section 3.1	Manufacturing and Production Difficulties	Scale-up of radiolabeled nanocarriers remains complex; alpha and Auger emitters require robust, cost-effective workflows under cGRPP/GMP.	[44,46,48]
Section 3.2	Regulatory and Reimbursement Barriers	Heterogeneity of regulatory frameworks and lack of harmonized reimbursement schemes slow clinical translation.	[27,46,48]
Section 3.3	Access and Availability of Radionuclides	Global dependence on limited infrastructure; recurrent shortages of 99Mo/99mTc linked to aging reactors and uneven cyclotron capacity.	[41,43,52,53,54,55,56,57,58,59,60]
Section 3.4	Integration into Clinical Practice	Difficulties in conducting large-scale trials due to supply constraints, radiation protection, and costs; the transition from preclinical to clinical is slow.	[42,46,48]
Section 3.5	Interdisciplinary Collaboration and Workforce Training	Implementation requires collaboration among chemists, clinicians, and regulators; workforce training gaps persist, especially in LMICs.	[33,41,48]
Section 3.6	Scientific and Technological Innovation	Progress depends on novel chelators, improved dosimetry, and safer delivery systems; critical nanodesign variables include size, stability, charge, and clearance.	[85,86,87,88,89,90,91]

**Figure 2:**
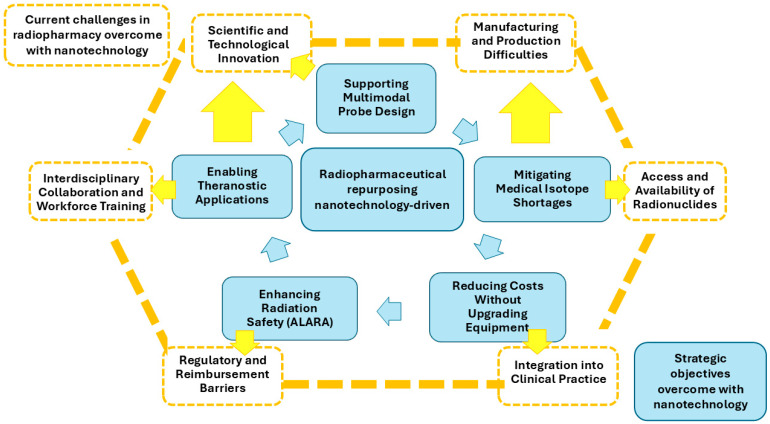
Visual map linking five nanotechnology-driven objectives for radiopharmaceutical repurposing (inner light blue ring) with six macro-challenges in radiopharmacy (outer orange ring). Colored arrows indicate direct impact in light blue; orange arrows indicate indirect or enabling effects. The figure illustrates where nanotechnology can most strongly contribute to translational progress, clinical integration, and resource optimization.

Figure 2 shows the mapping of the five nanotechnology objectives to the six major challenges in radiopharmaceutical sciences.

This approach is not limited to oncology. Nanotechnology can revive or optimize radiotracers for neurology, cardiology, infectious diseases, and inflammatory conditions, where

- High diagnostic sensitivity is critical (e.g., for amyloid plaques, infections, or microthrombi).
- Targeting specificity reduces false positives and off-target toxicity.
- Radiobiological safety margins are especially important (e.g., pediatric or vulnerable populations).
- Multimodal imaging can accelerate clinical decision-making.
- Despite its promise, nanotechnology cannot independently solve:
- Fragmented regulatory and reimbursement frameworks
- Insufficient radionuclide production infrastructure,
- Gaps in workforce training and multidisciplinary collaboration.

However, by enhancing the clinical, economic, and safety profiles of both established and repurposed radiopharmaceuticals, nanotechnology strengthens the case for regulatory approval, reimbursement, and broader adoption.

## 4. Functional Objectives of Nanotechnology-Enabled Drug Repurposing in Radiopharmacy

Nanotechnology offers a unique opportunity to overcome several intrinsic barriers associated with the repurposing of drugs in the field of radiopharmacy. Unlike conventional drug development, radiopharmaceuticals must satisfy complex physicochemical and biodistribution requirements that limit the feasibility of repositioning without formulation innovation. Here, we outline five key objectives that can be addressed by nanotechnology when applied to the repurposing of radiopharmaceutical agents.

### 4.1. Alleviating the Medical Isotope Shortage

The global supply of key medical isotopes, particularly ^99^Mo/^99m^Tc, remains a critical bottleneck for nuclear medicine [92]. Aging reactors, geopolitical factors, and limited production sites have led to recurrent shortages, which in turn jeopardize diagnostic workflows and patient care. In addition, emerging therapeutic radionuclides such as alpha-emitters (e.g., ^225^Ac, ^211^At) and Auger-electron emitters are produced in small quantities and require cost-intensive infrastructure, limiting their availability for routine clinical use.

Nanotechnology offers an innovative path to alleviate this shortage by enhancing the delivery efficiency and bioavailability of radionuclides, allowing smaller administered activities to achieve comparable or superior diagnostic and therapeutic effects [93,94]. By improving tumor accumulation and reducing off-target distribution, nanocarriers can minimize radiotracer waste and extend the clinical utility of existing isotope stocks. This is particularly valuable for high-demand agents such as ^99m^Tc-labeled tracers, which form the backbone of conventional nuclear medicine imaging. For example, lipid-based nanoparticles and polymeric micelles have been explored as carriers for ^99m^Tc complexes, achieving better pharmacokinetics and prolonged blood circulation times compared to free radiotracers. This allows for the use of reduced isotope doses without compromising image quality, thereby reducing the strain on isotope supply chains. Similarly, the encapsulation of therapeutic alpha-emitters (such as ^225^Ac) within nanoparticle matrices has demonstrated improved tumor uptake and decreased systemic toxicity, effectively increasing the therapeutic yield per unit of isotope administered [95,96,97]. Furthermore, radioisotope recycling strategies integrated with nanocarrier design—such as using nanoparticles with high chelation stability—can help reduce isotope loss during synthesis and labeling, which is another key factor in maximizing the efficiency of limited radionuclide resources.

Nanotechnology-driven optimization not only mitigates the current isotope shortage but also creates a foundation for repurposing older radiopharmaceuticals that were previously considered impractical due to high isotope demand or rapid clearance. With more efficient targeting and delivery, these compounds may regain clinical relevance in modern nuclear medicine. Several studies have illustrated the potential of nanotechnology to optimize radionuclide utilization and reduce reliance on scarce isotope supplies. One example involves ^99m^Tc-loaded liposomes, which have been engineered with PEGylated surfaces to enhance circulation time and tumor targeting. These formulations not only require lower initial activities to achieve high-quality imaging but also reduce radiotracer degradation during preparation and transport, improving the overall efficiency of ^99^Mo/^99m^Tc use. Another notable case is the nanoencapsulation of ^225^Ac within inorganic nanocarriers, such as lanthanide phosphate nanoparticles or silica-based matrices. These platforms provide robust chelation and minimize the release of radioactive daughters, which is a major safety and efficacy challenge in alpha therapy. By increasing retention at the tumor site and limiting systemic distribution, these nanocarriers effectively boost the therapeutic impact per administered dose, which is particularly valuable given the limited global production of ^225^Ac.

### 4.2. Reducing Costs Without Infrastructure Investment

By improving tracer stability, pharmacokinetics, and target-to-background ratios, nanotechnology may contribute to more efficient radiopharmaceutical procedures and better use of existing imaging infrastructure, complementing technological advances in scanner hardware by (i) reducing the administered activity through more efficient delivery, (ii) reducing tracer waste by improving formulation strategies and surface functionalization, while the intrinsic stability of radiolabeling remains dependent on the radionuclide–chelator pair, (iii) improving target-to-background ratios, which may enhance image quality and quantification in preclinical and early clinical studies, thereby potentially reducing the need for additional imaging sessions during development, and (iv) streamlining dosimetry and response monitoring in theranostic workflows. Although regulatory and reimbursement hurdles persist, nano-enabled dose-sparing and formulation robustness can improve cost-effectiveness profiles, strengthening the case for broader coverage and adoption [27,94,98,99,100].

The escalating costs of establishing and maintaining high-end hybrid imaging infrastructure (e.g., next-generation PET/CT or PET/MR) and scaling radioligand therapy programs have become a recognized barrier to equitable implementation of precision nuclear medicine. At the same time, reimbursement frameworks often lag behind scientific progress, creating economic bottlenecks that slow the clinical adoption of innovative agents [11,27,101]. Nanotechnology offers a complementary cost-containment path that does not depend on hardware upgrades: by improving delivery efficiency, radiochemical stability, and target selectivity, nanoformulations can reduce the activity needed to achieve diagnostic image quality or therapeutic efficacy, thereby lowering radionuclide consumption, minimizing waste, and decreasing the likelihood of repeat procedures.

From a diagnostic standpoint, numerous reviews document how ^99m^Tc-labeled nanoparticles, liposomes, polymeric micelles, and inorganic nanocarriers can be radiolabeled efficiently and tracked in vivo, often with higher in vivo stability and prolonged circulation than their small-molecule counterparts. This combination can translate into dose-sparing effects (less activity to reach the same contrast/noise level) and lower preparation losses, both of which reduce per-study isotope costs [94,98,100]. The same rationale applies to therapeutic nano-radiopharmaceuticals: by increasing tumor retention and reducing off-target deposition, nanoencapsulation can raise the therapeutic yield per unit of administered activity. Although most published work emphasizes biological performance rather than economic modeling, the ALARA-linked cost–benefit literature supports the logic that any strategy that reliably lowers administered activity and occupational exposure tends to be economically favorable when total costs (including protection, disposal, and repeat imaging/therapy) are accounted for [99,102,103].

Critically, improved formulation robustness (e.g., tighter chelation, reduced leaching, better recoil daughter retention in alpha-emitter nano-matrices) reduces batch failures, radiochemical rework, and disposal volumes, all of which carry direct and indirect costs [63,100]. These technical gains can strengthen health–economic arguments for coverage and reimbursement—an aspect repeatedly highlighted as a major barrier in radiotheranostics [27,104]. Moreover, by lowering per-procedure radionuclide needs, nano-enabled strategies can partially decompress strained isotope supply chains, indirectly stabilizing prices and availability in high-demand settings [11,94].

In sum, although nanotechnology cannot replace the need for regulatory clarity, expanded radionuclide production capacity, or new theragnostic centers, it can improve the cost-effectiveness envelope of both established and repurposed radiopharmaceuticals. This is achieved through dose sparing, fewer repeat scans, reduced waste, and better alignment with ALARA-driven economic optimization, ultimately lowering barriers to clinical integration without necessitating immediate capital investment in new imaging hardware [27,99,100,101].

### 4.3. Enabling ALARA Compliance Through Dose Minimization

Nanocarriers offer a promising avenue to enhance radiation protection in nuclear medicine by enabling more targeted delivery of radiopharmaceuticals, thus aligning with the ALARA principle. By reducing off-target accumulation, nanotechnology contributes to lower administered activities, minimizing unnecessary exposure for both patients and healthcare personnel.

Radiation safety has always been a central concern in the clinical application of radiopharmaceuticals, especially considering the dual imperative to optimize diagnostic or therapeutic outcomes while minimizing exposure to non-target tissues. The ALARA principle, widely adopted in nuclear medicine practice and regulation, emphasizes this balance by urging dose reductions wherever clinically and technically feasible. Nanotechnology introduces a transformative potential in this regard. Through improved pharmacokinetics and targeted biodistribution, nanocarriers can significantly reduce the off-target uptake of radioactive agents. For example, PEGylated nanoparticles or ligand-functionalized nanosystems can preferentially accumulate in tumor tissue or specific organs of interest, thereby limiting systemic radiation burden and sparing healthy tissues from unnecessary exposure [100]. A lower off-target accumulation directly correlates with a reduced need for high administered activity, which benefits both patient safety and operational radiation protection in clinical environments. For healthcare workers, particularly nuclear medicine technologists and radiopharmacists, this can translate into lower cumulative occupational doses, a key goal for long-term safety in high-throughput institutions [105]. Furthermore, nanoformulations may help decrease the biological half-life of radiopharmaceuticals in non-target organs, such as the liver or kidneys, by modifying the surface chemistry or adding biodegradable components that allow faster clearance. This optimization has been observed in nanoparticle-based agents labeled with ^64^Cu and ^68^Ga, which demonstrated reduced retention in critical organs without compromising tumor uptake [106,107].

In summary, nanotechnology-driven formulations offer concrete, mechanism-based strategies to comply with ALARA without sacrificing efficacy. This advancement is especially relevant for theragnostic agents and emerging alpha-emitting radiopharmaceuticals, where radiation burden to normal tissues must be carefully controlled.

### 4.4. Facilitating Theragnostic Design and Implementation

Nanotechnology enables the co-development of diagnostic and therapeutic agents within the same molecular architecture, optimizing biodistribution, targeting, and pharmacokinetics for theragnostic use. This integration can accelerate translation, improve patient stratification, and reduce development costs.

Theranostics, the combination of diagnostic and therapeutic capabilities within a single platform, has become a cornerstone of modern nuclear medicine. Theragnostic drug delivery systems aim to improve disease management by ensuring that the therapeutic agent and diagnostic tracer are co-delivered to the same target site, allowing for real-time monitoring of treatment efficacy and biodistribution. These systems are particularly significant in nuclear medicine, where radioactive isotopes can serve as both imaging markers and therapeutic agents [63]. However, the traditional development of matched diagnostic and therapeutic radiopharmaceuticals often requires complex parallel pipelines, demanding substantial time, infrastructure, and regulatory navigation. Nanotechnology provides an opportunity to streamline this process by integrating diagnostic and therapeutic functionalities into a unified nanocarrier system, thereby facilitating simultaneous or sequential applications for imaging and treatment. Nanostructures such as liposomes, dendrimers, polymersomes, and silica nanoparticles can be engineered to carry both diagnostic isotopes (e.g., ^68^Ga, ^89^Zr) and therapeutic payloads (e.g., ^177^Lu, ^90^Y), or to allow exchangeable labeling depending on clinical need [108,109]. This modularity enables the development of “plug-and-play” theragnostic agents with tunable properties that support patient-specific adaptation and stratification. In addition to radiolabeling flexibility, nanocarriers can prolong systemic circulation, enhance tumor accumulation via the enhanced permeability and retention (EPR) effect, and support controlled release mechanisms that further improve the therapeutic index. These features are particularly valuable in oncology, where precise temporal coordination between imaging and therapy may improve response monitoring and treatment planning [63]. Liposomal carriers exemplify this approach by being labeled with radionuclides such as technetium-99m, allowing for imaging, while simultaneously carrying chemotherapeutic agents like doxorubicin to target tumor tissues [110,111,112,113,114,115]. This combination enables clinicians to monitor treatment progress in real-time and adjust therapy accordingly. Rhenium isotopes, particularly rhenium-186 and rhenium-188, have been extensively investigated as theranostic agents due to their emission of both therapeutic beta particles and gamma rays suitable for imaging. However, despite promising preclinical and early clinical studies, their translation into widespread clinical use has been limited, and research activity in recent years has shifted toward other theranostic radionuclides [106,116,117,118,119,120,121,122,123,124,125,126,127,128,129,130,131]. When these isotopes are incorporated into liposomal systems alongside chemotherapeutic drugs, they facilitate targeted treatment of cancers such as head and neck tumors, demonstrating promising preclinical results. Moreover, the possibility to co-encapsulate radiosensitizers or immunomodulators together with radionuclides within the same nanoplatform expands the functional versatility of theragnostics. For instance, hybrid nanoformulations combining ^64^Cu or ^89^Zr imaging agents with chemotherapeutics or photothermal moieties are under evaluation for real-time treatment guidance and synergistic effects [132,133,134,135]. By leveraging multifunctional nanocarriers, drug repurposing efforts in radiopharmacy can be expanded beyond individual agent rescue to encompass integrated diagnostic-therapeutic paradigms, advancing precision nuclear medicine.

### 4.5. Designing Multimodal Imaging Probes

Multimodal agents are primarily designed to carry multiple diagnostic or imaging functionalities, often for comprehensive visualization or characterization, without necessarily including a therapeutic component. These may involve agents that can perform multi-modal imaging (e.g., PET, MRI, CT, optical imaging) to provide more detailed disease assessment, but are not inherently therapeutic. For instance, nanoparticles labeled with several isotopes for dual or multi-modal imaging (PET and MRI) exemplify multifunctionality. Nanotechnology offers an ideal foundation for engineering these probes due to its capacity for modular surface functionalization and co-loading of diverse agents. For example, a nanoparticle can be designed to carry both a radioisotope for PET imaging (e.g., ^64^Cu or ^89^Zr) and a near-infrared (NIR) fluorophore for intraoperative guidance [136,137]. In addition, the same nanoplatform may include a magnetic resonance (MR) contrast agent such as gadolinium or iron oxide, resulting in a tri-modal construct [138]. This integration facilitates synergistic diagnostics, as each modality offers unique spatiotemporal and physiological insights.

In the context of drug repurposing, nanotechnology can revitalize old molecular scaffolds by embedding them into platforms designed for multimodal detection. A chemotherapeutic or targeting ligand initially developed for another purpose may be conjugated to a radiolabeled nanoparticle, thereby transforming a non-imaging molecule into a diagnostic probe. An illustrative case involves the repurposing of iron oxide nanoparticles, traditionally used for MRI, which have been successfully radiolabeled with ^68^Ga, ^124^I, or ^64^Cu to enable PET/MR dual imaging [139,140]. This dual-modality system not only enhances lesion localization but also allows cross-validation of imaging signals, increasing clinical confidence and reducing false positives or negatives.

Beyond diagnostic enhancement, multifunctional nanoplatforms can be tailored to support theranostic applications, enabling both imaging and targeted therapy within a single nanosystem. One illustrative strategy involves the use of iron oxide nanoparticles as an MRI-visible core, functionalized with polyethylene glycol (PEG) chains to prolong systemic circulation. Surface modification with bifunctional chelators such as DOTA or NOTA enables radiolabeling with PET or SPECT isotopes, while simultaneously allowing the incorporation of therapeutic radionuclides like ^177^Lu or ^225^Ac [138]. This combinatorial design not only permits signal acquisition from nuclear and magnetic modalities but also facilitates therapeutic payload delivery guided by molecular targeting. Liposomal nanocarriers represent a particularly versatile platform for the co-encapsulation of diagnostic and therapeutic agents [63,141,142]. These systems can be engineered to incorporate multiple imaging labels within a single construct—for instance, gadolinium for MRI contrast, near-infrared fluorescent dyes for optical tracking, and radionuclides such as ^99m^Tc or ^64^Cu for SPECT or PET imaging. Importantly, the same liposomal core can be loaded post-formulation with chemotherapeutic agents such as doxorubicin, enabling a truly integrated theragnostic configuration. In other designs, liposomes have been functionalized to simultaneously deliver therapeutic radioisotopes (e.g., ^166^Ho), SPECT agents, and paramagnetic elements for MRI. This modularity in design not only allows for precise imaging across multiple modalities but also supports site-specific therapy and longitudinal treatment monitoring, reinforcing the clinical promise of multifunctional radiolabeled nanocarriers.

In sum, the design of multimodal imaging probes supported by nanotechnology represents a powerful convergence of imaging innovation and strategic drug repurposing. It embodies the principle of doing more with less—using existing molecules and infrastructure in novel ways to meet growing diagnostic complexity.

This framework highlights the strategic value of nanotechnology not only in developing novel radiopharmaceuticals but also in revisiting previously discarded or underutilized agents that can now meet clinical demands through formulation innovation. In the next section, we explore specific case examples that illustrate these principles in action.

## 5. Challenges in the Clinical Translation of Nanotechnology-Enabled Drug Repurposing in Radiopharmacy

### 5.1. Limited Clinical Translation Despite Extensive Preclinical Promise

Despite the significant number of promising preclinical studies demonstrating the utility of nanocarriers for radiopharmaceutical delivery, only a limited fraction of this research has reached clinical implementation. This disconnect between laboratory success and clinical application mirrors trends seen in other drug delivery fields [67,79,143,144]. The limitations observed are not merely downstream issues but reflect fundamental constraints that must be addressed from the earliest stages of formulation development.

Key barriers include difficulties in upscaling complex nanocarrier production, high costs associated with quality assurance and Good Manufacturing Practices (GMP)-compliant manufacturing, and prolonged registration timelines. Regulatory resistance is often exacerbated by the unique dual nature of radiopharmaceutical nanocarriers, which must satisfy nuclear, pharmaceutical, and toxicological requirements. Furthermore, intellectual property disputes, especially around the composition and use of repurposed drugs in novel delivery formats, can further hinder translational progress.

The integration of nanotechnology into drug repurposing strategies for radiopharmaceuticals demands a tailored and highly controlled approach. Unlike conventional formulations, the development of nanocarriers for radiopharmacy involves a complex interplay between the biological environment, the physicochemical behavior of the nanocarrier, and the nuclear characteristics of the radionuclide. Any misalignment between these components can undermine targeting efficiency, therapeutic effectiveness, and safety.

### 5.2. Technical and Biological Constraints of Radiopharmaceutical Nanocarriers

Nanocarriers intended for radiopharmaceutical repurposing must satisfy a demanding set of interdependent criteria to increase the likelihood of successful clinical translation. Among the most critical variables is particle size, which governs circulation time, tissue penetration, and clearance. Nanoparticles larger than ~400 nm are typically sequestered by the mononuclear phagocyte system, leading to rapid clearance and limited accumulation at disease sites [145]. Conversely, extremely small particles (<35 nm) can behave similarly to antibodies, favoring passive targeting. To exploit the EPR effect in tumors or inflamed tissues, optimal carrier size often falls between 10 and 500 nm, with molecular weights under 80 kDa [146]. Techniques such as sonoporation may extend the usable size range for larger particles by temporarily enhancing vascular permeability [147].

Stability of the carrier and controlled release of the radiotracer are additional prerequisites. Premature or burst release of the radionuclide at non-target sites results in non-specific radiation exposure, reduced therapeutic efficacy, and increased systemic toxicity [148]. The nanocarrier must retain the radiopharmaceutical during circulation, withstand recoil energies—particularly important for alpha emitters—and ensure release precisely at the target site, synchronized with the physical half-life of the radionuclide to maximize imaging or therapeutic efficacy [149]. Particles that aggregate into structures exceeding 5 µm in diameter may pose embolic risk and must therefore be avoided.

Surface charge also plays a key role in biodistribution. A moderately negative surface charge can reduce opsonization and clearance by the reticuloendothelial system, prolonging circulation time [150,151]. However, excessive surface negativity may promote hepatic accumulation, leading to off-target exposure and toxicity, necessitating careful modulation of surface properties [152].

Physiological and toxicological acceptability is critical. All components of the nanocarrier—core materials, surface modifiers, and active targeting ligands such as antibodies or peptides—must be biocompatible, immunologically inert, and ideally recognized as safe (GRAS) substances. The reformulated product should not enhance the inherent toxicity of the repurposed radiotracer. For instance, liposomal encapsulation of doxorubicin, though designed to reduce systemic toxicity, led to an increased incidence of hand-foot syndrome due to extended circulation time [152]. Formulations that fail to deliver their payload efficiently must be rapidly eliminated to minimize radiation burden and avoid image artifacts.

When multiple agents are co-encapsulated—such as a radionuclide and a chemotherapeutic—they must be pharmacologically compatible and exhibit aligned pharmacokinetics. Comprehensive preclinical characterization of the nanocarrier’s biodistribution, metabolism, and excretion is indispensable before clinical translation [153]. Additional formulation challenges emerge when targeting tissues with physiological barriers such as the blood–brain barrier (BBB). In such cases, nanocarriers must be engineered to cross these barriers while maintaining compatibility with the decay profile and biological action of the radiotracer [149,154].

These considerations should not be viewed merely as barriers, but rather as foundational criteria that shape the rational design and development of repurposed radiopharmaceuticals using nanotechnology. Addressing them early in the process is crucial to unlocking the full translational potential of this approach. Ultimately, the design of nanotechnology-enabled drug repurposing systems in radiopharmacy must be biologically rational, technically feasible, and pharmaceutically safe. Their successful translation requires early integration of nuclear medicine considerations into the formulation process, along with rigorous assessment of each component’s behavior within the complex biological environment.

### 5.3. Regulatory and Translational Outlook

The convergence of drug repurposing strategies and nanotechnology has given rise to a new class of radiopharmaceuticals—repurposed nanoradiotheranostics—that challenge traditional regulatory paradigms. These agents originate from known molecules such as radionuclides, targeting vectors, or therapeutic compounds, but acquire new properties through encapsulation, conjugation, or co-formulation with nanomaterials. Depending on the strategy employed, repurposing may involve (1) new indications (repositioning); (2) altered routes or formulations (reformulation); or (3) combinatory strategies with complementary agents or modalities. Each of these approaches can significantly alter the safety, pharmacokinetics, and therapeutic profile of the original agent, especially when the molecular architecture becomes a hybrid nanosystem.

This transformation has both functional and regulatory consequences. By embedding repurposed radiopharmaceuticals in nanocarriers, the resulting construct frequently qualifies as a new chemical entity. In some cases, nanotechnology has enabled the rescue of previously discontinued agents by improving their biodistribution, enhancing stability, or reducing toxicity, which may revitalize molecules with otherwise limited clinical utility. Such repositioned nanotheranostics—particularly those integrating both imaging and therapeutic functionalities—demand an updated evaluation framework encompassing novel physicochemical, biological, and radiological features [155,156]. Illustrative advances have also been reported in complementary imaging modalities such as MRI and fluorescence imaging. In MRI, emerging nanoplatforms are being investigated as alternatives to conventional gadolinium-based agents, aiming to reduce potential toxicity while maintaining or enhancing contrast efficiency [157,158]. Likewise, nanotechnology-based materials have been developed for fluorescence imaging, offering improved photostability, higher quantum yield, and opportunities for multimodal integration [159,160,161].

From a quality and safety standpoint, these nanostructured platforms require extensive characterization beyond traditional radiopharmaceutical standards. Parameters such as size, surface charge, composition, in vivo stability, drug release kinetics, and immune interaction profiles must be documented. Additional nanoparticle-specific requirements include assessments of colloidal stability in physiological media, long-term organ accumulation, interaction with serum proteins, and the presence of nanoparticle degradation products. These considerations are compounded in radionanotheranostic systems, where the radiolabeling process itself can affect nanoparticle structure, and the recoil energy from radioactive decay may compromise formulation integrity [155,156].

Dosimetric evaluation is further complicated by altered biodistribution and pharmacokinetics induced by nanocarriers. Regulatory agencies increasingly demand bridging studies or new preclinical data to establish whether previously approved dosimetry and toxicity data remain applicable. Comparability protocols—used to assess equivalence between the original and reformulated agents—remain inconsistently defined across jurisdictions, especially in cases involving multifunctional constructs [155,156].

While these molecular-level innovations raise fundamental questions regarding safety and efficacy, regulatory frameworks have not evolved at the same pace. Most agencies continue to extrapolate existing guidelines for conventional radiopharmaceuticals or general nanomedicines, which do not fully account for the dual complexity of radionanotheranostic products. This misalignment generates uncertainty for developers, slows down translation, and complicates dossier preparation [155,156].

A major obstacle is regulatory classification. Depending on its dominant mechanism of action and therapeutic purpose, a single agent may be variably classified as a drug, device, biologic, or combination product. For instance, a PET-labeled nanoparticle carrying both a cytotoxic payload and an antibody fragment might fall under concurrent oversight by radiopharmaceutical, biologics, and nanomedicine regulators, leading to duplicative or even contradictory requirements. These classification inconsistencies are particularly burdensome in multinational development efforts where harmonized approaches are crucial [155,156].

Furthermore, personalized and stratified applications—such as those guided by companion diagnostics or molecular imaging—add further complexity. Regulatory approval for these agents often requires robust biomarker validation, adaptive clinical trial designs, and individualized dosimetric models, especially in cases where the nanocarrier is expected to overcome previous therapeutic limitations [155,156].

Overlaying these molecular and regulatory dimensions is a third layer of complexity: the incorporation of digital and algorithmic components. AI-assisted imaging tools, software-based dosimetry systems, and adaptive treatment planning platforms are becoming essential to the clinical implementation of multifunctional nanoradiopharmaceuticals. These technologies—often classified as software-as-a-medical-device (SaMD)—must be independently validated for analytical performance, clinical utility, and data integrity, particularly when they influence decisions such as dose calculation, target selection, or therapy scheduling. Harmonizing their evaluation with the radiopharmaceutical product itself remains a largely unresolved challenge [155,156].

In parallel, ethical and environmental considerations must be addressed. The long-term fate of radiolabeled nanomaterials in biological systems and the environment remains poorly understood, and the role of algorithmic decision-making in clinical pathways raises concerns about transparency and accountability [155,156].

Given these intersecting challenges, there is growing momentum to establish risk-based, harmonized regulatory frameworks that account for the multifunctionality, hybrid nature, and repurposed origins of these agents. Regulatory initiatives such as the Food and Drug Administration (FDA) Emerging Technology Program and European Medicines Agency (EMA) Innovation Task Force are beginning to address these gaps, as are transnational efforts through regulatory science consortia like the Nanomedicine Characterization Laboratory (NCL) and the European Nanomedicine Characterisation Laboratory (EUNCL), which offer standardized preclinical assessment protocols for nanoconjugates [155,156].

Illustrative failures of promising constructs further highlight the urgency for reform. Radiolabeled nanoparticles combining PET tracers with chemotherapeutic or MRI-active agents have stalled due to unresolved issues in scale-up reproducibility, dual labeling stability, or classification ambiguity. To accelerate the bench-to-bedside trajectory of repurposed nanoradiotheranostics, regulatory frameworks must evolve to accommodate scientific convergence—across disciplines, modalities, and technologies—without compromising safety, efficacy, or transparency.

Several recent initiatives illustrate how regulatory agencies are beginning to adapt to the unique challenges of nanotechnology-enabled radiopharmaceuticals. The European Medicines Agency (EMA) Innovation Task Force (ITF) offers free, early-stage, informal briefing meetings to developers—including academics and SMEs—on regulatory, technical, and scientific concerns, well before formal submissions are prepared [162]. At a structural level, the EMA has established a dedicated Expert Group on Nanomedicines and generated multiple reflection papers on liposomal and nanocolloidal products, supported by international collaboration (including FDA, Health Canada, PMDA/Australia) to harmonize scientific evaluation of next-generation nanomedicines [163].

On the U.S. side, the FDA’s Nanotechnology Core Facility (Nanocore) has developed ASTM-standardized methods for liposome characterization and nanomaterial testing, facilitating regulatory clarity in quality and safety assessment [164]. Additionally, an exploratory IND (eIND) was used to conduct a phase 0 microdosing trial of ^188^Re-liposome in cancer patients, demonstrating the practical application of regulatory flexibility for novel nano-radiopharmaceuticals [165].

To complement the discussion on harmonization, Table 3 summarizes representative regulatory initiatives and international frameworks that have directly or indirectly shaped the development of nanoradiopharmaceuticals.

### 5.4. Current Limitations and Research Priorities

Nanotechnology-enabled drug repurposing in radiopharmacy faces several constraints: limited and heterogeneous clinical evidence; incomplete and non-standardized physicochemical–radiochemical characterization; insufficient quantitative imaging and dosimetry methods adapted to nanoformulations; and regulatory, manufacturing, and reimbursement barriers. Additional challenges include variability introduced by reformulation into nanocarriers, immune system interactions, lack of open and reproducible datasets, and gaps in intellectual property protection that may reduce commercial incentives.

Short-term priorities include harmonizing minimum characterization protocols, developing quantitative imaging–dosimetry workflows tailored to nanostructures, implementing bridging studies for comparability, establishing predictive biomarkers for patient selection, and promoting open data practices. In the medium term, adaptive multicenter trials, integration of multi-omics and imaging data, modular GMP manufacturing platforms, consensus guidelines for formulation changes, and long-term safety studies will be essential. These actions can accelerate translation while ensuring safety, reproducibility, and clinical relevance for nano-enabled repurposed radiopharmaceuticals.

## 6. Conclusions and Future Directions

The convergence of nanotechnology and radiopharmaceutical science is opening new frontiers in precision medicine, particularly through the strategic repurposing of known agents. By reformulating diagnostic and therapeutic compounds within multifunctional nanocarriers, it becomes possible to unlock novel applications, overcome biological and pharmacokinetic limitations, and enhance clinical performance. This paradigm not only optimizes the value of existing pharmacological knowledge but also accelerates innovation in a cost- and time-efficient manner.

Yet, the translational path of such advanced constructs—radionanotheranostics—remains hindered by scientific, technical, and regulatory complexities. The inherent multifunctionality of these agents challenges conventional evaluation frameworks, demanding updated methodologies for safety assessment, dosimetry, pharmacokinetics, and clinical validation. Moreover, the integration of auxiliary technologies—such as AI-guided image analysis and software-assisted planning—calls for new regulatory paradigms capable of addressing hybrid products that blur traditional disciplinary and jurisdictional boundaries.

As the field matures, there is an urgent need for harmonized regulatory approaches that accommodate the uniqueness of these constructs without compromising safety or efficacy. Risk-based classification strategies, tailored preclinical protocols, and robust comparability criteria will be essential to navigate the complexity introduced by nanostructuring and molecular repurposing. Equally important is the development of regulatory science tools—such as standardized characterization platforms, predictive modeling, and advanced in vivo imaging techniques—that can streamline the transition from bench to bedside.

Looking forward, the successful integration of repurposed radiopharmaceuticals into nanotechnology-based platforms will rely on sustained interdisciplinary collaboration. Chemists, radiopharmacists, clinicians, materials scientists, regulatory experts, and data scientists must work together to build not only smarter agents, but also smarter evaluation systems. Advances in modular design, in silico modeling, and systems-level pharmacology will further support the evolution of these technologies into safe, effective, and clinically meaningful tools.

Ultimately, radionanotheranostics represent more than the sum of their parts. They embody a shift toward integrated, adaptive, and patient-centered care, where diagnosis and therapy are not only co-localized but also co-designed. The promise of this approach lies not just in technological novelty but in its capacity to redefine therapeutic success through precision, personalization, and translational intelligence.

## Figures and Tables

**Table 1:** Comparison of the present review with representative recent publications on nanotechnology in radiopharmacy or radiotheranostics. The table highlights differences in scope, methodological approach, and emphasis on regulatory or translational aspects. Unlike most previous works, this review frames nanotechnology as a strategic enabler for radiopharmaceutical drug repurposing—covering both clinically approved and advanced preclinical agents—maps functional objectives to macro-level challenges in the field, and addresses applications beyond oncology for a multidisciplinary audience.

Reference	Primary Focus	Scope Beyond Oncology	Methodological Approach	Regulatory/Translational Emphasis	Unique Contribution
Jalilian et al., 2022 [14]	Overview of nanosized targeted radiopharmaceuticals	Limited	Descriptive listing of nanoformulations	Low	Frames nanotechnology as a strategic tool for radiopharmaceutical repurposing, cross-mapping with macro-level challenges
Trujillo-Nolasco et al., 2021 [15]	Alpha-emitter nanoradiopharmaceuticals	No	Focused on alpha emitters	Moderate	Broader radionuclide coverage (β-, α-, Auger), functional objectives beyond oncology
Klain et al., 2021 [16]	Functional imaging in thyroid cancer	No	Disease-specific	Low	Multi-disease scope, including neurology, cardiology, infectious and inflammatory disorders
Bodei et al., 2022 [27]	Radiotheranostics in oncology	No	Clinical translation focus	High	Integration of theranostic strategies with nano-enabled repurposing objectives
This review	Strategic objectives for nanotechnology-enabled drug repurposing in radiopharmacy	Yes	Conceptual framework + functional mapping	High	Novel cross-walk between nano-enabled objectives and macro-challenges; regulatory + translational focus; multi-specialty audience

**Table 3:** Representative regulatory and harmonization initiatives relevant to nanoradiopharmaceuticals.

Reference	Material/Technique Type	Regulatory Relevance
[155]. Giammarile et al., *Lancet Oncol.* (2024)	Theranostics (general)	Production and regulatory issues: Discusses the challenges of production and the regulatory issues of theranostics. This is a direct and crucial reference on the topic.
[156]. Gawne et al., *Nat Rev Mater.* (2023)	Nanotheranostics (general)	Clinical translation and challenges: Analyzes the obstacles in the clinical translation of nanotheranostics, addressing regulatory and development challenges.
[162]. EMA-Innovation Task Force (ITF)	Regulatory framework (EMA)	EMA regulatory guidance: A document describing meetings with the European Medicines Agency’s (EMA) Innovation Task Force. This is a direct reference to an official regulatory framework.
[163]. Bartlett et al., *AAPS J.* (2015)	Nanomaterials in drug products	PQRI Workshop on Nanomaterials: A report summarizing risk management and experience with nanomaterials in drug products. This is a crucial reference on safety and regulatory evaluation.
[164]. FDA NCTR Research Highlights	Regulatory framework (FDA)	FDA research advancements: An FDA report that may include studies on the characterization and toxicity of nanomaterials, which supports regulatory decisions. This is a direct reference to a regulatory body.
[165]. Chang et al., *Int. J. Mol. Sci.* (2021)	Nanotargeted 188Re-Liposomes	Translation of research: A specific case study of the clinical translation of a nanoradiopharmaceutical. Highlights the need for a regulatory approach for these types of products.

## Data Availability

Data sharing is not applicable.

## Abbreviations

The following abbreviations are used in this manuscript:

ALARA	As Low As Reasonably Achievable
CT	Computed Tomography
DOTA	1,4,7,10-tetraazacyclododecane-1,4,7,10-tetraacetic acid
EMA	European Medicines Agency
EPR	Enhanced Permeability and Retention
EUNCL	European Nanomedicine Characterisation Laboratory
FDA	Food and Drug Administration
GMP	Good Manufacturing Practices
MRI	Magnetic Resonance Imaging
NCL	Nanomedicine Characterization Laboratory
NOTA	1,4,7-Triazacyclononane-1,4,7-triacetic acid
PD	Pharmacodynamics
PEG	Polyethylene glycol
PET	Positron Emission Tomography
PK	Pharmacokinetics
SaMD	Software-as-a-medical-device
SPECT	Single Photon Emission Tomography

## References

[1] Cañellas C.O., Salgueiro M.J., Zubillaga M. (2017). Radiofármacos: Del Laboratorio al Paciente.

[2] Strosberg J., El-Haddad G., Wolin E., Hendifar A., Yao J., Chasen B., Mittra E., Kunz P.L., Kulke M.H., Jacene H. (2017). NETTER-1 Trial Investigators. Phase 3 Trial of ^177^Lu-Dotatate for Midgut Neuroendocrine Tumors. N. Engl. J. Med..

[3] Sartor O., de Bono J., Chi K.N., Fizazi K., Herrmann K., Rahbar K., Tagawa S.T., Nordquist L.T., Vaishampayan N., El-Haddad G. (2021). VISION Investigators. Lutetium-177-PSMA-617 for Metastatic Castration-Resistant Prostate Cancer. N. Engl. J. Med..

[4] Hofman M.S., Emmett L., Sandhu S., Iravani A., Joshua A.M., Goh J.C., Pattison D.A., Tan T.H., Kirkwood I.D., Ng S. (2021). [177Lu]Lu-PSMA-617 versus cabazitaxel in patients with metastatic castration-resistant prostate cancer (TheraP): A randomised, open-label, phase 2 trial. Lancet.

[5] Miederer M. (2022). Alpha emitting nuclides in nuclear medicine theranostics. Nuklearmedizin.

[6] Poty S., Francesconi L.C., McDevitt M.R., Morris M.J., Lewis J.S. (2018). Alpha-Emitters for Radiotherapy: From Basic Radiochemistry to Clinical Studies-Part 1. J. Nucl. Med..

[7] Poty S., Francesconi L.C., McDevitt M.R., Morris M.J., Lewis J.S. (2018). α-Emitters for Radiotherapy: From Basic Radiochemistry to Clinical Studies-Part 2. J. Nucl. Med..

[8] Borgna F., Haller S., Rodriguez J.M.M., Ginj M., Grundler P.V., Zeevaart J.R., Köster U., Schibli R., van der Meulen N.P., Müller C. (2022). Combination of terbium-161 with somatostatin receptor antagonists-a potential paradigm shift for the treatment of neuroendocrine neoplasms. Eur. J. Nucl. Med. Mol. Imaging.

[9] Funkhouser J. (2002). Reinventing pharma: The theranostic revolution. Curr. Drug Discov..

[10] Kuge Y., Shiga T., Tamaki N. (2016). Perspectives on Nuclear Medicine for Molecular Diagnosis and Integrated Therapy.

[11] Scott A.M., Zeglis B.M., Lapi S.E., Scott P.J.H., Windhorst A.D., Abdel-Wahab M., Giammarile F., Paez D., Jalilian A., Knoll P. (2024). Trends in nuclear medicine and the radiopharmaceutical sciences in oncology: Workforce challenges and training in the age of theranostics. Lancet Oncol..

[12] McGuireWoods Radiopharmaceutical Industry Update: Q4 2024–Q1 2025. https://www.mcguirewoods.com/client-resources/alerts/2025/4/radiopharmaceutical-industry-update-q4-q1-2024-2025.

[13] Lapi S.E., Scott P.J.H., Scott A.M., Windhorst A.D., Zeglis B.M., Abdel-Wahab M., Baum R.P., Buatti J.M., Giammarile F., Kiess A.P. (2024). Recent advances and impending challenges for the radiopharmaceutical sciences in oncology. Lancet Oncol..

[14] Jalilian A.R., Ocampo-García B., Pasanphan W., Sakr T.M., Melendez-Alafort L., Grasselli M., Lugao A.B., Yousefnia H., Dispenza C., Janib S.M. (2022). IAEA Contribution to Nanosized Targeted Radiopharmaceuticals for Drug Delivery. Pharmaceutics.

[15] Trujillo-Nolasco M., Morales-Avila E., Cruz-Nova P., Katti K.V., Ocampo-García B. (2021). Nanoradiopharmaceuticals Based on Alpha Emitters: Recent Developments for Medical Applications. Pharmaceutics.

[16] Klain M., Zampella E., Nappi C., Nicolai E., Ambrosio R., Califaretti E., Lamartina L., Schlumberger M., Deandreis D., Salvatore D. (2021). Advances in Functional Imaging of Differentiated Thyroid Cancer. Cancers.

[17] Roy I., Krishnan S., Kabashin A.V., Zavestovskaya I.N., Prasad P.N. (2022). Transforming Nuclear Medicine with Nanoradiopharmaceuticals. ACS Nano.

[18] Li X., Wang C., Tan H., Cheng L., Liu G., Yang Y., Zhao Y., Zhang Y., Li Y., Zhang C. (2016). Gold nanoparticles-based SPECT/CT imaging probe targeting for vulnerable atherosclerosis plaques. Biomaterials.

[19] Su T., Wang Y.B., Han D., Wang J., Qi S., Gao L., Shao Y.H., Qiao H.Y., Chen J.W., Liang S.H. (2017). Multimodality Imaging of Angiogenesis in a Rabbit Atherosclerotic Model by GEBP11 Peptide Targeted Nanoparticles. Theranostics.

[20] Pérez-Medina C., Binderup T., Lobatto M.E., Tang J., Calcagno C., Giesen L., Wessel C.H., Witjes J., Ishino S., Baxter S. (2016). In Vivo PET Imaging of HDL in Multiple Atherosclerosis Models. JACC Cardiovasc. Imaging.

[21] Nahrendorf M., Hoyer F.F., Meerwaldt A.E., van Leent M.M.T., Senders M.L., Calcagno C., Robson P.M., Soultanidis G., Pérez-Medina C., Teunissen A.J.P. (2020). Imaging Cardiovascular and Lung Macrophages With the Positron Emission Tomography Sensor ^64^Cu-Macrin in Mice, Rabbits, and Pigs. Circ. Cardiovasc. Imaging.

[22] Ahmadi A., Thorn S.L., Alarcon E.I., Kordos M., Padavan D.T., Hadizad T., Cron G.O., Beanlands R.S., DaSilva J.N., Ruel M. (2015). PET imaging of a collagen matrix reveals its effective injection and targeted retention in a mouse model of myocardial infarction. Biomaterials.

[23] Gomes-da-Silva N.C., Xavier-de-Britto I., Soares M.A.G., Yoshihara N.M.A., Ilem Özdemir D., Ricci-Junior E., Fechine P.B.A., Alencar L.M.R., Henriques M.d.G.M.d.O., Barja-Fidalgo T.C. (2025). Nanostructured Lipoxin A4: Understanding Its Biological Behavior and Impact on Alzheimer’s Disease (Proof of Concept). Pharmaceutics.

[24] Underwood C., van Eps A.W., Ross M.W., Laverman P., van Bloois L., Storm G., Schaer T.P. (2012). Intravenous technetium-99m labelled PEG-liposomes in horses: A safety and biodistribution study. Equine Vet. J..

[25] de Assis D.N., Mosqueira V.C., Vilela J.M., Andrade M.S., Cardoso V.N. (2008). Release profiles and morphological characterization by atomic force microscopy and photon correlation spectroscopy of 99mTechnetium-fluconazole nanocapsules. Int. J. Pharm..

[26] de Assis D.N., Araújo R.S., Fuscaldi L.L., Fernandes S.O.A., Mosqueira V.C.F., Cardoso V.N. (2018). Biodistribution of free and encapsulated ^99m^Tc-fluconazole in an infection model induced by Candida albicans. Biomed. Pharmacother..

[27] Bodei L., Herrmann K., Schöder H., Scott A.M., Lewis J.S. (2022). Radiotheranostics in oncology: Current challenges and emerging opportunities. Nat. Rev. Clin. Oncol..

[28] Schwenck J., Sonanini D., Cotton J.M., Rammensee H.G., la Fougere C., Zenber L., Pichler B.J. (2023). Advances in PET imaging of Cancer. Nat. Rev. Cancer.

[29] Hanahan D., Weinberg R.A. (2011). Hallmarks of cancer: The next generation. Cell.

[30] Hanahan D. (2022). Hallmarks of cancer: New dimensions. Cancer Discov..

[31] Hanahan D., Weinberg R.A. (2000). The hallmarks of cancer. Cell.

[32] Herrmann K.S.M., Lewis J.S., Solomon S.B., McNeil B.J., Baumann M., Gambhir S.S., Hricak H., Weissleder R. (2020). Radiotheranostics: A roadmap for future development. Lancet Oncol..

[33] Weber W.A., Anderson G., Badawi R.D., Barthel H., Bengel F., Bodei L., Buvat I., DiCarli M., Graham M.M., Grimm J. (2020). The future of nuclear medicine, molecular imaging, and theranostics. J. Nucl. Med..

[34] Radvanyi P., Villain J. (2017). The discovery of radioactivity. Comptes Rendus Phys..

[35] Hevesy G. (1923). The absorption and translocation of lead by plants: A contribution to the application of the method of radioactive indicators in the investigation of the change of substance in plants. Biochem. J..

[36] Myers W.G. (1996). Georg Charles de Hevesy: The father of nuclear medicine. J. Nucl. Med. Technol..

[37] Lawrence J., Tuttle L., Scott K., Connor C. (1940). Studies on neoplasms with the aid of radioactive phosphorus. I. The total phosphorus metabolism of normal and leukemic mice. J. Clin. Investig..

[38] Tuttle L.W., Erf L.A., Lawrence J.H. (1941). Studies on neoplasms with the aid of radioactive phosphorus. II. The phosphorus metabolism of the nucleoprotein, phospholipid and acid soluble fractions of normal and leukemic mice. J. Clin. Investig..

[39] Chamberlain R.H., Low-Beer B.V.A., Thomas C.C. (1950). The clinical use of radioactive isotopes. Science.

[40] Beierwaltes W.H., Johnson P.C., Solari A.J. (1957). Clinical Use of Radioisotopes.

[41] Czernin J., Calais J. (2022). How many theranostics centers will we need in the united states?. J. Nucl. Med..

[42] Jadvar H., Chen X., Cai W., Mahmood U. (2018). Radiotheranostics in cancer diagnosis and management. Radiology.

[43] Cutler C.S., Bailey E., Kumar V., Schwarz S.W., Bom H.S., Hatazawa J., Paez D., Orellana P., Louw L., Mut F. (2021). Global Issues of Radiopharmaceutical Access and Availability: A Nuclear Medicine Global Initiative Project. J. Nucl. Med..

[44] Gillings N., Hjelstuen O., Ballinger J., Behe M., Decristoforo C., Elsinga P., Ferrari V., Peitl P.K., Koziorowski J., Laverman P. (2021). Guideline on current good radiopharmacy practice (cGRPP) for the small-scale preparation of radiopharmaceuticals. EJNMMI Radiopharm. Chem..

[45] Baum R.P., Kulkarni H.R. (2012). THERANOSTICS: From Molecular Imaging Using Ga-68 Labeled Tracers and PET/CT to Personalized Radionuclide Therapy—The Bad Berka Experience. Theranostics.

[46] Turner J.H. (2018). Recent advances in theranostics and challenges for the future. Br. J. Radiol..

[47] Poot A.J., Lam M.G.E.H., van Noesel M.M. (2020). The Current Status and Future Potential of Theranostics to Diagnose and Treat Childhood Cancer. Front. Oncol..

[48] Herrmann K., Giovanella L., Santos A., Gear J., Kiratli P.O., Kurth J., Denis-Bacelar A.M., Hustinx R., Patt M., Wahl R.L. (2022). Joint EANM, SNMMI and IAEA enabling guide: How to set up a theranostics centre. Eur. J. Nucl. Med. Mol. Imaging.

[49] Urbain J.L., Scott A.M., Lee S.T., Buscombe J., Weston C., Hatazawa J., Kinuya S., Singh B., Haidar M., Ross A. (2023). Theranostics Radiopharmaceuticals: A Universal Challenging Educational Paradigm in Nuclear Medicine. J. Nucl. Med..

[50] Fahey F.H., Goodkind A., MacDougall R.D., Oberg L., Ziniel S.I., Cappock R., Callahan M.J., Kwatra N., Treves S.T., Voss S.D. (2017). Operational and Dosimetric Aspects of Pediatric PET/CT. J. Nucl. Med..

[51] Hendrikse H., Kiss O., Kunikowska J., Wadsak W., Decristoforo C., Patt M. (2022). EANM position on the in-house preparation of radiopharmaceuticals. Eur. J. Nucl. Med. Mol. Imaging.

[52] International Atomic Energy Agency Database of Cyclotrons for Radionuclide Production. https://nucleus.iaea.org/sites/accelerators/Pages/Cyclotron.aspx.

[53] International Atomic Energy Agency (2011). Disposal of radioactive waste. IAEA Safety Standards Series No. SSR-5, Vienna.

[54] Hricak H., Abdel-Wahab M., Atun R., Lette M.M., Paez D., Brink J.A., Donoso-Bach L., Frija G., Hierath M., Holmberg O. (2021). Medical imaging and nuclear medicine: A Lancet Oncology Commission. Lancet Oncol..

[55] Giammarile F., Delgado Bolton R.C., El-Haj N., Freudenberg L.S., Herrmann K., Mikhail M., Morozova O., Orellana P., Pellet O., Estrada L.E. (2021). Changes in the global impact of COVID-19 on nuclear medicine departments during 2020: An international follow-up survey. Eur. J. Nucl. Med. Mol. Imaging.

[56] Delgado Bolton R.C., Calapaquí Terán A.K., Erba P.A., Giammarile F. (2021). Medical imaging in times of pandemic: Focus on the cornerstones of successful imaging. Eur. J. Nucl. Med. Mol. Imaging.

[57] Paez D., Mikhail-Lette M., Gnanasegaran G., Dondi M., Estrada-Lobato E., Bomanji J., Vinjamuri S., El-Haj N., Morozova O., Alonso O. (2022). Nuclear Medicine Departments in the Era of COVID-19. Semin. Nucl. Med..

[58] International Atomic Energy Agency Research Reactor Database (RRDB). https://nucleus.iaea.org/rrdb/#/home.

[59] International Atomic Energy Agency (2003). Manual for Reactor Produced Radioisotopes, IAEA-TECDOC-1340, Vienna. https://www-pub.iaea.org/MTCD/publications/PDF/te_1340_web.pdf.

[60] International Atomic Energy Agency (2019). Predisposal Management of Radioactive Waste from the Use of Radioactive Material in Medicine, Industry, Agriculture, Research and Education. IAEA Safety Standards Series No. SSG-45, Vienna. https://www.iaea.org/publications/11087/predisposal-management-of-radioactive-waste-from-the-use-of-radioactive-material-in-medicine-industry-agriculture-research-and-education.

[61] (2017). International Atomic Energy Agency. Management of Radioactive Waste from the Use of Radionuclides in Medicine, IAEA-TECDOC-1805, Vienna.

[62] International Atomic Energy Agency Radiation Protection and Safety in Medical Uses of Ionizing Radiation, IAEA Safety Standards Series No. SSG-46, Vienna 2018. https://www.iaea.org/publications/11102/radiation-protection-and-safety-in-medical-uses-of-ionizing-radiation.

[63] Kleynhans J., Grobler A.F., Ebenhan T., Sathekge M.M., Zeevaart J.R. (2018). Radiopharmaceutical enhancement by drug delivery systems: A review. J. Control Release.

[64] Filzen L.M., Ellingson L.R., Paulsen A.M., Hung J.C. (2017). Potential Ways to Address Shortage Situations of ^99^Mo/^99m^Tc. J. Nucl. Med. Technol..

[65] Skliarova H., Cisternino S., Cicoria G., Marengo M., Palmieri V. (2018). Innovative Target for Production of Technetium-99m by Biomedical Cyclotron. Molecules.

[66] Chess R. (1998). Economics of drug delivery. Pharm. Res..

[67] Allen T.M., Cullis P.R. (2004). Drug delivery systems: Entering the mainstream. Science.

[68] Mishra N., Pant P., Porwal A., Jaiswal J., Samad M.A., Tiwari S. (2016). Targeted drug delivery: A review. Am. J. Pharmatech. Res..

[69] Sercombe L., Veerati T., Moheimani F., Wu S.Y., Sood A.K., Hua S. (2015). Advances and challenges of liposome assisted drug delivery. Front. Pharmacol..

[70] Zylberberg C., Matosevic S. (2016). Pharmaceutical liposomal drug delivery: A review of new delivery systems and a look at the regulatory landscape. Drug Deliv..

[71] Batista C.A., Larson R.G., Kotov N.A. (2015). Nonadditivity of nanoparticle interaction. Science.

[72] Sahil K., Akanksha M., Premjeet S., Bilandi A. (2011). Kapoor BMicrosphere: A review. Int. J. Res. Pharm. Chem..

[73] Valliant J.F. (2016). A bridge not too far: Linking disciplines through molecular imaging probes. J. Nucl. Med. Tech..

[74] Duvall W.L., Croft L.B., Ginsberg E.S., Einstein A.J., Guma K.A., George T., Henzlova M.J. (2011). Reduced isotope dose and imaging time with a high-efficiency CZT SPECT camera. J. Nucl. Cardiol..

[75] Galea R., Ross C., Wells R.G. (2014). Reduce, reuse and recycle: A green solution to Canada’s medical isotope shortage. Appl. Radiat. Isot..

[76] Hoedl S.A., Updegraff W.D. (2015). The production of medical isotopes without nuclear reactors or uranium enrichment. Sci. Glob. Secur..

[77] Kelkar S.S., Reineke T.M. (2011). Theranostics: Combining imaging and therapy. Bioconjug. Chem..

[78] Kim T.H., Lee S., Chen X. (2013). Nanotheranostics for personalized medicine. Expert. Rev. Mol. Diagn..

[79] Kaul A., Chaturvedi S., Attri A., Kalra M., Mishra A.K. (2016). Targeted theranostic liposomes: Rifampicin and ofloxacin loaded pegylated liposomes for theranostic application in mycobacterial infections. RSC Adv..

[80] Wang S.J., Lin W.J., Chen M.N., Chi C.S., Chen J.T., Ho W.L., Hsieh B.T., Shen L.H., Tsai Z.T., Ting G. (1998). Intratumoral injection of rhenium-188 microspheres into an animal model of hepatoma. J. Nucl. Med..

[81] Wunderlich G., Pinkert J., Stintz M., Kotzerke J. (2005). Labelling and biodistribution of different particle materials for radioembolization therapy with 188Re. Appl. Radiat. Isot..

[82] Blankespoor S.C., Wu X., Kalki J.K., Brown H.R., Tang H.R., Cann C.E., Hasegawa B.H. (1996). Attenuation correction of SPECT using x-ray CT on an Emission-Transmission CT System: Myocardial perfusion assessment. IEEE Trans. Nucl. Sci..

[83] Shao Y., Cherry S.R., Farahani K., Meadors K., Siegel S.B., Silwerman R.W., Marsden P.K. (1997). Simultaneous PET and MR imaging. Phys. Med. Biol..

[84] Beyer T., Townsend W.D., Burn T., Kinahan P.E., Charron M., Roddy R., Jerin J., Young J., Byars L., Nutt R. (2000). A combined PET/CT scanner for clinical oncology. J. Nucl. Med..

[85] Townsend W.D. (2008). Dual-modality imaging: Combining anatomy and function. J. Nucl. Med..

[86] Czernin J., Allen-Auerbach M., Schelbert H.R. (2007). Improvements in cancer staging with PET/CT: Literature-based evidence as of September 2006. J. Nucl. Med..

[87] Judenhofer M.S., Wehrl H.F., Newport D.F., Catana C., Siegel S.B., Becker M., Thielscher A., Kneilling M., Lichy M.P., Eichner M. (2008). Simultaneous PET-MRI: A new approach for functional and morphological imaging. Nat. Med..

[88] Cheon J., Lee J.H. (2008). Synergistically integrated nanoparticles as multimodal probes for nanobiotechnology. ACC. Chem. Res..

[89] Wehrl H.F., Sauter A.W., Judenhofer M.S., Pichler B.J. (2010). Combined PET/MR imaging—Technology and applications. Technol. Cancer Res. Treat..

[90] Delso G., Fust S., Jakoby B., Ladebeck R., Ganter C., Nekolla S.G., Schwaiger M., Ziegler S.I. (2011). Performance measurements of the Siemens mMR integrated whole-body PET/MR scanner. J. Nucl. Med..

[91] Tan I.C., Darne C., Lu Y., Yan S., Smith A., Rasmussen J., Azhdarinia A., Sevick E. (2012). Hybrid fluorescence, PET, and CT for small animal imaging. J. Nucl. Med..

[92] Committee on State of Molybdenum-99 Production and Utilization and Progress Toward Eliminating Use of Highly Enriched Uranium, Nuclear and Radiation Studies Board, Division on Earth and Life Studies, National Academies of Sciences, Engineering, and Medicine (2016). Molybdenum-99 for Medical Imaging.

[93] Man F., Gawne P.J., de Rosales R.T.M. (2019). Nuclear imaging of liposomal drug delivery systems: A critical review of radiolabelling methods and applications in nanomedicine. Adv. Drug Deliv. Rev..

[94] Mushtaq S., Bibi A., Park J.E., Jeon J. (2021). Recent Progress in Technetium-99m-Labeled Nanoparticles for Molecular Imaging and Cancer Therapy. Nanomaterials.

[95] Toro-González M., Akingbesote N., Bible A., Pal D., Sanders B., Ivanov A.S., Jansone-Popova S., Popovs I., Benny P., Perry R. (2024). Development of ^225^Ac-doped biocompatible nanoparticles for targeted alpha therapy. J. Nanobiotechnol..

[96] Toro-González M., Dame A.N., Mirzadeh S., Rojas J.V. (2020). Encapsulation and retention of ^225^Ac, ^223^Ra, ^227^Th, and decay daughters in zircon-type gadolinium vanadate nanoparticles. Radiochim. Acta.

[97] Woodward J., Kennel S.J., Stuckey A., Osborne D., Wall J., Rondinone A.J., Standaert R.F., Mirzadeh S. (2011). LaPO4 nanoparticles doped with actinium-225 that partially sequester daughter radionuclides. Bioconjug. Chem..

[98] Trusova V., Karnaukhov I., Zelinsky A., Borts B., Ushakov I., Sidenko L., Gorbenko G. (2024). Radiolabeling of bionanomaterials with technetium 99m: Current state and future prospects. Nanomedicine.

[99] Engström A., Isaksson M., Javid R., Lundh C., Båth M. (2021). A case study of cost-benefit analysis in occupational radiological protection within the healthcare system of Sweden. J. Appl. Clin. Med. Phys..

[100] Pellico J., Gawne P.J., de Rosales R.T.M. (2021). Radiolabelling of nanomaterials for medical imaging and therapy. Chem. Soc. Rev..

[101] Pomykala K.L., Würker M., Herrmann K. (2023). Tackling the Last Mile: A Major Component to Successfully Establish Radioligand Therapy. J. Nucl. Med..

[102] Aerts A., Eberlein U., Holm S., Hustinx R., Konijnenberg M., Strigari L., van Leeuwen F.W.B., Glatting G., Lassmann M. (2021). EANM position paper on the role of radiobiology in nuclear medicine. Eur. J. Nucl. Med. Mol. Imaging.

[103] Blanc-Béguin F., Damien P., Floch R., Kerleguer K., Hennebicq S., Robin P., Salaün P.Y., Le Roux P.Y. (2022). Radiation exposure to nuclear medicine technologists performing a V/Q PET: Comparison with conventional V/Q scintigraphy, [^18^F]FDG PET and [^68^Ga]Ga DOTATOC PET procedures. Front. Med..

[104] Aboagye E.O., Barwick T.D., Haberkorn U. (2023). Radiotheranostics in oncology: Making precision medicine possible. CA Cancer J. Clin..

[105] Thakral P., Sen I., Simecek J., Marx S., Kumari J., Kumar S., Tandon P., Dureja S., Pant V. (2020). Radiation Exposure to the Nuclear Medicine Personnel During Preparation and Handling of ^213^Bi-Radiopharmaceuticals. J. Nucl. Med. Technol..

[106] Rosenblum D., Joshi N., Tao W., Karp J.M., Peer D. (2018). Progress and challenges towards targeted delivery of cancer therapeutics. Nat. Commun..

[107] Ehlerding E.B., Grodzinski P., Cai W., Liu C.H. (2018). Big Potential from Small Agents: Nanoparticles for Imaging-Based Companion Diagnostics. ACS Nano.

[108] Aminolroayaei F., Shahbazi-Gahrouei D., Shahbazi-Gahrouei S., Rasouli N. (2021). Recent nanotheranostics applications for cancer therapy and diagnosis: A review. IET Nanobiotechnol..

[109] Gupta D., Roy P., Sharma R., Kasana R., Rathore P., Gupta T.K. (2024). Recent nanotheranostic approaches in cancer research. Clin. Exp. Med..

[110] Goins B., Bao A., Phillips W.T. (2017). Techniques for loading technetium-99m and rhenium-186/188 radionuclides into preformed liposomes for diagnostic imaging and radionuclide therapy. Methods Mol. Biol..

[111] Belhaj-Tayeb H., Briane D., Vergote J., Kothan S., Leger G., Bendada S.E., Tofighi M., Tamgac F., Cao A., Moretti J.L. (2003). In vitro and in vivo study of 99mTc-MIBI encapsulated in PEG-liposomes: A promising radiotracer for tumour imaging. Eur. J. Nucl. Med. Mol. Imaging.

[112] Goins B., Klipper R., Rudolph A.S., Phillips W.T. (1994). Use of technetium-99m-liposomes in tumour imaging. J. Nucl. Med..

[113] Dams E.T., Oyen W.J., Boerman O.C., Storm G., Laverman P., Kok P.J., Buijs W.C., Bakker H., van der Meer J.W., Corsten F.H. (2000). 99mTc-PEG liposomes for the scintigraphic detection of infection and inflammation: Clinical evaluation. J. Nucl. Med..

[114] 115 Dagar S., Krishnadas A., Rubinstein I., Blend M.J., Onyuksel H. (2003). VIP grafted sterically stabilized liposomes for targeted imaging of breast cancer: In Vivo studies. J. Control. Release.

[115] Kleiter M.M., Yu D., Mohammadian L.A., Niehaus N., Spasojevic I., Sanders L., Viglianti B.L., Yarmolenko P.S., Hauck M., Petry N.A. (2006). A tracer dose of technetium-99m-labeled liposomes can estimate the effect of hyperthermia on intratumoral doxil extravasation. Clin. Cancer Res..

[116] Chen M.H., Chang C.H., Chang Y.J., Chen L.C., Yu C.Y., Wu Y.H., Lee W.C., Yeh C.H., Lin F.H., Lee T.W. (2010). MicroSPECT/CT imaging and pharmacokinetics of 188Re-(DXR)-liposome n human colorectal adenocarcinoma-bearing mice. Anticancer Res..

[117] Tsai C.C., Chang C.H., Chen L.C., Chang Y.J., Lan K.L., Wu Y.H., Hsu C.W., Liu I.H., Ho C.L., Lee W.C. (2011). Biodistribution and pharmacokinetics of 188Re-liposomes and their comparative therapeutic efficacy with 5-fluorouracil in a C26 colonic peritoneal carcinomatosis mice. Int. J. Nanomed..

[118] Liu C.M., Chang C.H., Chang Y.J., Hsu C.W., Chen L.C., Chen H.L., Ho C.L., Yu C.Y., Chang T.J., Chiang T.C. (2010). Preliminary evaluation of acute toxicity of 188Re-BMEDA-liposome in rats. J. Appl. Toxicol..

[119] Chi-Mou L., Chia-Che T., Chia-Yu Y., Wan-Chi L., Chung-Li H., Tsui-Jung C., Chih-Hsien C., Te-Wei L. (2013). Extended acute toxicity study of 188Re-liposome in rats. J. Appl. Toxicol..

[120] Hsu W.H., Liu S.Y., Chang Y.J., Chang C.H., Ting G., Lee T.W. (2014). The PEGylated liposomal doxorubicin improves the delivery and therapeutic efficiency of 188Re-Liposome by modulating phagocytosis in C26 murine colon carcinoma tumour mode. Nucl. Med. Biol..

[121] Chang C.H., Liu S.Y., Chi C.W., Yu H.L., Chang T.J., Tsai T.H., Lee T.W., Chen Y.J. (2015). External beam radiotherapy synergizes 188Re-liposome against human oesophageal cancer xenograft and modulates 188Re-liposome pharmacokinetics. Int. J. Nanomed..

[122] Allard E., Hindre F., Passirani C., Lemaire L., Lepareur N., Noiret N., Menei P., Benoit J.P. (2008). 188Re-loaded lipid nanocapsules as a promising radiopharmaceutical carrier for internal radiotherapy of malignant gliomas. Eur. J. Nucl. Med. Mol. Imaging.

[123] Wang S.X., Bao A., Herrera S.J., Phillips W.T., Goins B., Santoyo C., Miller F.R., Otto R.A. (2008). Intraoperative 186Re-liposome radionuclide therapy in a head and neck squamous cell carcinoma xenograft positive surgical margin model. Clin. Cancer Res..

[124] Zavaleta C.L., Goins B.A., Bao A., McManus L.M., McMahan C.A., Phillips W.T. (2008). Imaging of 186Re-liposome therapy in ovarian cancer xenograft model of peritoneal carcinomatosis. J. Drug Target..

[125] Chang Y.J., Yu C.Y., Hsu C.W., Lee W.C., Chen S.J., Chang C.H., Lee T.W. (2012). Molecular imaging and therapeutic efficacy of 188Re-(DXR)-liposome-BNN in AR42J pancreatic tumour-bearing mice. Oncol. Rep..

[126] Chen L.C., Wu Y.H., Liu I.H., Ho C.L., Lee W.C., Chang C.H., Lan K.L., Ting G., Lee T.W., Shien J.H. (2012). Pharmacokinetics, dosimetry and comparative efficacy of 188Re-liposome and 5-Fu in a CT26-luc lung-metastatic mice model. Nucl. Med. Biol..

[127] Phillips W.T., Goins B., Bao A., Vargas D., Guttierez J.E., Trevino A., Miller J.R., Henry J., Zuniga R., Vecil G. (2012). Rhenium-186 liposomes as convection-enhanced nanoparticle brachytherapy for treatment of glioblastoma. Neuro Oncol..

[128] Liu C.M., Lee W.C., Yu C.Y., Lan K.L., Chang C.H., Ting G., Lee T.W. (2012). Comparison of the therapeutic efficacy of 188Rhenium-liposomes and liposomal doxurbicin in a 4T1 murine orthotopic breast cancer model. Oncol. Rep..

[129] Shen Y.A., Lan K.L., Chang C.H., Lin L.T., He C.L., Chen P.H., Lee T.W., Lee Y.J., Chuang C.M. (2016). Intraperitoneal (188)Re-liposome delivery switches ovarian cancer metabolism from glycolysis to oxidative phosphorylation and effectively controls ovarian tumour growth in mice. Radiother. Oncol..

[130] Cao J., Wang Y., Yu J., Xia J., Zang C., Yin D., Häfeli U.O. (2004). Preparation and radiolabeling of surface-modified magnetic nanoparticles with rhenium-188 for magnetic targeted radiotherapy. J. Magn. Magn. Mater..

[131] Liang S., Wang Y., Zang C., Liu X. (2006). Synthesis of amino-modified magnetite nanoparticles coated with Hepama-1 and radiolabeled with 188Re for bio-magnetically targeted radiotherapy. J. Radioanal. Nucl. Chem..

[132] Chen H., Zhang W., Zhu G., Xie J., Chen X. (2017). Rethinking cancer nanotheranostics. Nat. Rev. Mater..

[133] Kashyap B.K., Singh V.V., Solanki M.K., Kumar A., Ruokolainen J., Kesari K.K. (2023). Smart Nanomaterials in Cancer Theranostics: Challenges and Opportunities. ACS Omega.

[134] Wang B., Hu S., Teng Y., Chen J., Wang H., Xu Y., Wang K., Xu J., Cheng Y., Gao X. (2024). Current advance of nanotechnology in diagnosis and treatment for malignant tumors. Signal Transduct. Target. Ther..

[135] Azimizonuzi H., Ghayourvahdat A., Ahmed M.H., Kareem R.A., Zrzor A.J., Mansoor A.S., Athab Z.H., Kalavi S. (2025). A state-of-the-art review of the recent advances of theranostic liposome hybrid nanoparticles in cancer treatment and diagnosis. Cancer Cell Int..

[136] Karageorgou M.A., Bouziotis P., Stiliaris E., Stamopoulos D. (2023). Radiolabeled Iron Oxide Nanoparticles as Dual Modality Contrast Agents in SPECT/MRI and PET/MRI. Nanomaterials.

[137] Abou D.S., Thorek D.L., Ramos N.N., Pinkse M.W., Wolterbeek H.T., Carlin S.D., Beattie B.J., Lewis J.S. (2013). 89Zr-labeled paramagnetic octreotide-liposomes for PET-MR imaging of cancer. Pharm. Res..

[138] Pellico J., Ruiz-Cabello J., Herranz F. (2023). Radiolabeled iron oxide nanomaterials for multimodal nuclear imaging and positive contrast MRI. ACS Appl. Nano Mater..

[139] Shi X., Sun Y., Shen L. (2022). Preparation and in vivo imaging of a novel potential α_v_β_3_ targeting PET/MRI dual-modal imaging agent. J. Radioanal. Nucl. Chem..

[140] Glaus C., Rossin R., Welch M.J., Bao G. (2010). In Vivo evaluation of 64Cu-labeled magnetic nanoparticles as a dual-modality PET/MR imaging agent. Bioconjug. Chem..

[141] Li S., Goins B., Zhang L., Bao A. (2012). A novel multifunctional theranostic liposome drug delivery system: Construction, characterization, and multimodality MR, Near-infrared fluorescent and nuclear imaging. Bioconjug. Chem..

[142] Zielhuis S.W., Seppenwoolde J.H., Mateus V.A., Bakker C.J., Krijger G.C., Storm G., Zonnenberg B.A., van het Schip A.D., Koning G.A., Nijsen J.F. (2006). Lanthanide-loaded liposomes for multimodality imaging and therapy. Cancer Biother. Radiopharm..

[143] Zang J., Gu F.X., Chan J.M., Wang A.Z., Langer R.S., Farokhzad O.C. (2008). Nanoparticles in medicine: Therapeutic applications and developments. Clin. Pharm. Therap..

[144] Sawant R.R., Torchillin V.P. (2012). Challenges in development of targeted liposomal therapeutics. AAPS J..

[145] Litzinger D.C., Buiting A.M.J., Van Rooijen N., Huang L. (1994). Effect of liposome size on the circulation time and intraorgan distribution of amphipathic poly(ethylene glycol)-containing liposomes. Biochim. Biophys. Acta.

[146] Torchilin V. (2011). Tumor delivery of macromolecular drugs based on the EPR effect. Adv. Drug Deliv. Rev..

[147] Couvreur P. (2013). Nanoparticles in drug delivery: Past, present and future. Adv. Drug Deliv. Rev..

[148] Theek B., Baues M., Ojha T., Möckel D., Veettil S.K., Steitz J., van Bloois L., Storm G., Kiessling F., Lammers T. (2016). Sonoporation enhances liposome accumulation and penetration in tumors with low EPR. J. Control. Release.

[149] Petrak K. (2006). Essential properties of drug-targeting delivery systems. Drug Discov. Today.

[150] Allen T.M., Cullis P.R. (2013). Liposomal drug delivery systems: From concept to clinical application. Adv. Drug Deliv. Rev..

[151] Meada H., Nakamura H., Fang J. (2013). The EPR effect for macromolecular drug delivery to solid tumors: Improvement of tumor uptake, lowering of systemic toxicity and distinct tumor imaging in vivo. Adv. Drug Deliv. Rev..

[152] Uriely B., Jeffers S., Isacson R., Kutch K., Wei-Tsao D., Yehoshua Z., Libson E., Muggia F.M., Lasic D.D. (1995). Liposomal doxorubicin: Antitumor activity and unique toxicities during two complementarty phase I studies. J. Clin. Oncol..

[153] Yamashita F., Hashida M. (2013). Pharmacokinetic considerations for targeted drug delivery. Adv. Drug Deliv. Rev..

[154] Pardridge W.M. (2005). The blood-brain barrier: Bottleneck in brain drug development. NeuroRx.

[155] Giammarile F., Paez D., Zimmermann R., Cutler C.S., Jalilian A., Korde A., Knoll P., Ayati N., Lewis J.S., Lapi S.E. (2024). Production and regulatory issues for theranostics. Lancet Oncol..

[156] Gawne P.J., Ferreira M., Papaluca M., Grimm J., Decuzzi P. (2023). New Opportunities and Old Challenges in the Clinical translation of Nanotheranostics. Nat. Rev. Mater..

[157] Kostevšek N., Cheung C.C.L., Serša I., Kreft M.E., Monaco I., Comes Franchini M., Vidmar J., Al-Jamal W.T. (2020). Magneto-Liposomes as MRI Contrast Agents: A Systematic Study of Different Liposomal Formulations. Nanomaterials.

[158] Di Gregorio E., Rosa E., Ferrauto G., Diaferia C., Gallo E., Accardo A., Terreno E. (2023). Development of cationic peptide-based hydrogels loaded with iopamidol for CEST-MRI detection. J. Mater. Chem. B.

[159] Bijoch Ł., Włodkowska U., Kasztelanic R., Pawłowska M., Pysz D., Kaczmarek L., Lapkiewicz R., Buczyński R., Czajkowski R. (2023). Novel Design and Application of High-NA Fiber Imaging Bundles for In Vivo Brain Imaging with Two-Photon Scanning Fluorescence Microscopy. ACS Appl. Mater. Interfaces.

[160] Gallo E., Diaferia C., Balasco N., Sibillano T., Roviello V., Giannini C., Vitagliano L., Morelli G., Accardo A. (2021). Fabrication of fluorescent nanospheres by heating PEGylated tetratyrosine nanofibers. Sci. Rep..

[161] Khalid A., Tomljenovic-Hanic S. (2024). Emerging Fluorescent Nanoparticles for Non-Invasive Bioimaging. Molecules.

[162] EMA-Innovation Task Force Briefing Meetings. https://www.ema.europa.eu/en/human-regulatory-overview/research-development/innovation-task-force-briefing-meetings.

[163] Bartlett J.A., Brewster M., Brown P., Cabral-Lilly D., Cruz C.N., David R., Eickhoff W.M., Haubenreisser S., Jacobs A., Malinoski F. (2015). Summary report of PQRI Workshop on Nanomaterial in Drug Products: Current experience and management of potential risks. AAPS J..

[164] FDA 2021 NCTR Research Highlights and Accomplishments. https://www.fda.gov/about-fda/nctr-publications/2021-nctr-research-highlights.

[165] Chang C.H., Chang M.C., Chang Y.J., Chen L.C., Lee T.W., Ting G. (2021). Translating Research for the Radiotheranostics of Nanotargeted ^188^Re-Liposome. Int. J. Mol. Sci..

